# Method of invitation and geographical proximity as predictors of NHS Health Check uptake

**DOI:** 10.1093/pubmed/fdu092

**Published:** 2014-11-26

**Authors:** Christopher Gidlow, Naomi Ellis, Jason Randall, Lisa Cowap, Graham Smith, Zafar Iqbal, Jagdish Kumar

**Affiliations:** 1 Centre for Research in Sport, Health and Exercise, Staffordshire University, Stoke on Trent ST4 2DF, UK; 2 Institute for Environment, Sustainability and Regeneration, Staffordshire University, Stoke on Trent ST4 2DF, UK; 3 NHS Stoke on Trent, Public Health Directorate, Civic Centre, ST4 1HH, UK; 4 Havering Public Health, Mercury House, Essex RM1 3DW, UK

**Keywords:** chronic disease, population-based and preventative services, screening

## Abstract

**Background:**

Uptake of NHS Health Checks remains below the national target. Better understanding of predictors of uptake can inform targeting and delivery. We explored invitation method and geographical proximity as predictors of uptake in deprived urban communities.

**Methods:**

This observational cohort study used data from all 4855 individuals invited for an NHS Health Check (September 2010–February 2014) at five general practices in Stoke-on-Trent, UK. Attendance/non-attendance was the binary outcome variable. Predictor variables included the method of invitation, general practice, demographics, deprivation and distance to Health Check location.

**Results:**

Mean attendance (61.6%) was above the city and national average, but varied by practice (47.5–83.3%; *P* < 0.001). Telephone/verbal invitations were associated with higher uptake than postal invitations (OR = 2.87, 95% CI = 2.26–3.64), yet significant practice-level variation remained. Distance to Health Check was not associated with attendance. Increasing age (OR = 1.04, 95% CI = 1.03–1.04), female gender (OR = 1.48, 95% CI = 1.30–1.68) and living in the least deprived areas (OR = 1.59, 95% CI = 1.23–2.05) were all independent positive predictors of attendance.

**Conclusions:**

Using verbal or telephone invitations should be considered to improve Health Check uptake. Other differences in recruitment and delivery that might explain remaining practice-level variation in uptake warrant further exploration. Geographical proximity may not be an important predictor of uptake in urban populations.

## Introduction

Cardiovascular diseases (CVDs) account for one-third of deaths globally, and remain the dominant non-communicable disease cluster.^1^ In 2009, the Department of Health in England introduced NHS Health Checks,^2^ a national vascular screening programme now supported by Public Health England (PHE), NHS England (NHSE), the National Institute for Health and Care Excellence (NICE) and the Local Government Association (LGA).^3^ The Health Check programme aims to identify people at risk of developing preventable illness including heart disease, stroke, diabetes and kidney disease, to facilitate discussion and action around prevention and risk management. Everyone in England between the ages of 40 and 74 without an existing chronic condition should be invited for a Health Check once every 5 years. Checks are typically completed in primary care by Practice Nurses, Health Care Support Workers or General Practitioners, with subsequent treatment, monitoring or signposting to local services as appropriate.

Initial modelling of cost-effectiveness predicted significant public health benefits in terms of chronic disease prevention (annual prevention of 1600 heart attacks and strokes, 650 premature deaths, over 4000 diabetes cases) and more effective disease management through early detection.^4^ However, this was based on a 75% uptake. Since implementation, uptake has fallen short of this level with recent national estimates of 49%.^5^ Understanding which people do not take up the Health Check invitation, the underlying reasons and ways to improve programme reach has, therefore, become a priority.^3^

Published research on NHS Health Checks has identified some patterns in uptake that are often observed in screening programmes. These include lower uptake in men, people at the younger end of the target age range and people with better health profiles.^6^ Associations between deprivation and uptake have been less consistent. Higher deprivation has been linked with lower uptake,^7^ which is consistent with evidence from other screening programmes,^8^ whereas some have reported higher uptake in more deprived areas^9^ or no relationship.^10^

Geographical access is a recognized barrier to heath service utilization. Evidence from the other screening programmes^11,12^ and health promotion initiatives^13^ suggest that those with worse physical access are less likely to use such services. So far, this has not been explored for Health Checks.

Practice size has also been implicated, with speculation that smaller practices are able to offer better quality and continuity of care, which might promote uptake of such preventive health opportunities.^6,10^ The method of invitation, however, is largely unexplored. Data from large CVD screening programmes indicate that uptake through traditional postal invitation is low.^14–16^ Although research and evaluation around improving the standard Health Check invitation letter is ongoing in some localities, letters remain the dominant approach. The role of telephone or opportunistic verbal invitations is unclear.

There is a recognized need for robust, controlled trials to improve the evidence base around Health Checks, but ‘the responsible authorities do not have the luxury of being able to wait for long-term trials before deciding what to do’ (p. 4);^17^ Many local authorities commission evaluations to ensure that implementation is evidence-informed. We report data from a sample of general practices in Stoke-on-Trent, a relatively deprived urban area of the UK, where overall uptake at the time of this study (50.2%) approximately in line with the national average (49%). Our aim was to explore the role of geographical proximity (or access) and method of invitation in predicting uptake of NHS Health Checks in deprived urban communities.

## Methods

### Settings and participants

Five general practices in Stoke-on-Trent, UK, participated. Practices provided anonymized data on all patients who had been invited for a Health Check between September 2010 and February 2014 (*n* = 4855). All practices were located in urban areas (although some patients resided in rural neighbourhoods), with practice lists that ranged from 2800 to over 10 000.

### Study design and procedures

This observational cohort study involved analysis of routinely gathered data from patients invited for a Health Check. All 53 practices in the city were given the opportunity to register their interest via an email invitation. Of the 18 practices that responded, 16 could be followed up and 5 were selected that could comply with the study requirements in the specified timeframe (e.g. run searches to identify Health Check attenders/non-attenders and associated socio-demographic data; facilitate other parts of the project).^18^ Practices were asked to run searches to provide data on uptake and a range of predictor variables for all patients invited for NHS Health since implementation.

### Measures

#### Uptake

The primary outcome measure was attendance (or ‘uptake’) of the Health Check versus non-attendance.

#### Socio-demographic

Age, sex and ethnicity were captured. Socio-economic position was estimated using income deprivation from the Index of Multiple Deprivation (IMD) 2010^19^ according to the Lower Super Output Area (LSOA) of residence. Income deprivation was favoured as a more direct estimate of material deprivation, rather than using the overall IMD, which includes indicators related to the living environment and barriers to housing and services. Geographical access to the general practice was explored using a separate variable.

#### Geographical proximity to Health Check location

Postcodes were used to explore physical distance (or geographical access) as a possible predictor of uptake. Using Geographical Information Systems (GIS) each general practice where Health Checks were conducted was geocoded and all attenders/non-attenders were mapped using the centre-point of their residential postcode. Distance from residential postcode to practice along the road and pedestrian network was then estimated for each participant. Although less accurate than using address location (data not available), most postcode areas were urban and, therefore, relatively small. As such, distance from postcode area centre points to general practices provided a good estimate of participant home-general practice distance. Moreover, using distance along the road and pedestrian network, rather than the more common Euclidean (or straight line) distance, took into account area differences in street connectivity and walkability.^20^

#### Data analysis

Data were screened for obvious errors, anomalies and outliers. In addition to boxplots, *z*-scores were produced for continuous variables (e.g. distance to Health Check location) and all values more than 3.29 SD from the mean were checked and exclusion considered on a case-by-case basis. Chi-squared tests were used to compare differences between attender and non-attender groups. A two-stage binary logistic regression analysis was used to explore predictors of Health Check attendance (where non-attendance = 0, attendance = 1). In Step 1, factors related to the Health Check process were entered (invitation method, practice). In Step 2, factors relating to the individual were entered to determine if they added further explanatory power to the model (age, sex, deprivation, geographical proximity).

## Results

### Sample profile and overall uptake

Data were available for 4855 individuals invited for a Health Check (Table 1). There were slightly more women than men (53.1 versus 46.9%), a mean age of 53.4 ± 9.1 years, and highest representation of those aged 45–50 years. Somewhat typical of the local population, almost 40% resided in areas within the most income-deprived quintile of national rankings and most were classified as British/White British (88.3%). Half lived within 2 km of their general practice.

**Table 1:** Sample characteristics

	*All*		*Attenders*		*Non-attenders*		*Difference^a^*	
	**n**	*%*	**n**	*%*	**n**	*%*	*χ^2^*	**P**
Total	4855		2989	61.57	1866	38.43		
Sex							34.73	<0.001
Male	2278	46.90	1304	43.63	974	52.20		
Female	2573	53.00	1685	56.37	888	47.59		
Total	4851	99.90	2989	100.00	1862	99.79		
Missing	4	0.10	0	0.00	4	0.21		
Age category (year)							132.76	<0.001
<45	778	16.02	448	14.99	330	17.72		
45–49.9	1065	21.94	551	18.43	514	27.60		
50–54.9	709	14.60	443	14.82	266	14.29		
55–59.9	840	17.30	506	16.93	334	17.94		
60–64.9	670	13.80	440	14.72	230	12.35		
65–69.9	470	9.68	340	11.38	130	6.98		
70+	319	6.57	261	8.73	58	3.11		
Total	4851	99.92	2989	100.00	1862	100.00		
Missing	4	0.08	0	0.00	4	0.00		
Income deprivation quintile							17.983	<0.001
1 (most deprived)	1723	37.71	1053	37.37	670	38.26		
2	477	10.81	324	12.41	153	8.25		
3	1081	24.96	644	24.52	437	25.67		
4	949	16.35	560	15.59	389	17.58		
5 (least deprived)	613	9.91	405	10.00	208	9.75		
Total	4843	99.75	2986	99.90	1857	99.52		
Missing	12	0.25	3	0.10	9	0.48		
Ethnicity^b^							0.769	0.380
White/White British	4285	88.26	2767	92.57	1518	81.35		
Mixed	20	0.41	15	0.50	5	0.27		
Asian/Asian British	133	2.74	80	2.68	53	2.84		
Black/Black British	24	0.49	15	0.50	9	0.48		
Other	7	0.14	3	0.10	4	0.21		
Total	4469	92.05	2880	96.35	1589	85.16		
Missing	386	7.95	109	3.65	277	14.84		
Distance to practice (km)							0.478	0.924
<1 km	1458	30.25	903	30.46	555	29.92		
1–1.9 km	923	19.15	573	19.33	350	18.87		
2–3.9 km	1609	33.38	984	33.19	625	33.69		
≥4 km	830	17.22	505	17.03	325	17.52		
Total	4820	99.28	2965	99.20	1855	99.41		
Missing	35	0.72	24	0.80	11	0.59		

^a^Difference between attender and non-attender groups.

^b^Due to the small number of participants per cell (i.e. <5), χ^2^ test used to compare White/White British with all other categories combined.

### Patterns in uptake

Mean Health Check uptake (or attendance) was 61.6%, but varied by practice (range 47.5–83.3%; *χ*^2^ = 336.9_(4)_, *P* < 0.001). Demographic differences in uptake were in the expected direction, with higher attendance in older age groups and women (Table 1), but no significant differences by ethnic group. There was an overall effect for deprivation, but given the likely clustering of participants around practices, apparent patterns were treated with caution and further explored in regression.

**Fig. 1:**
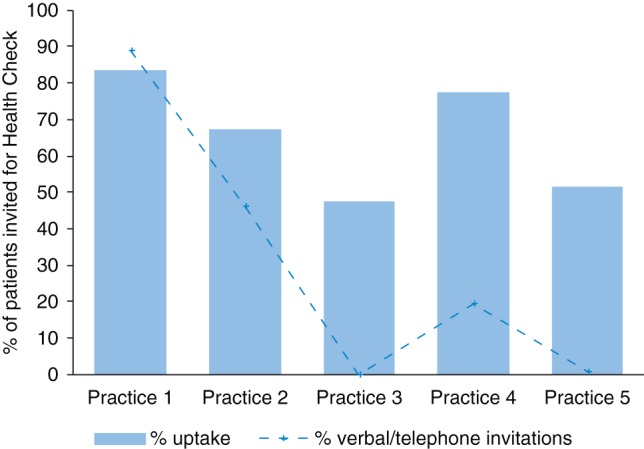
Within-practice variation in uptake and the proportion of Health Check invitations that were verbal/telephone (rather than letter only).

Most individuals were invited for a Health Check through letters (72%), but this varied between practices (Fig. 1). Using telephone/verbal invitations, either alone or in combination with the letter, was linked with significantly higher attendance (*χ*^2^ = 316.24_(1)_, *P* < 0.001).

**Fig. 2:**
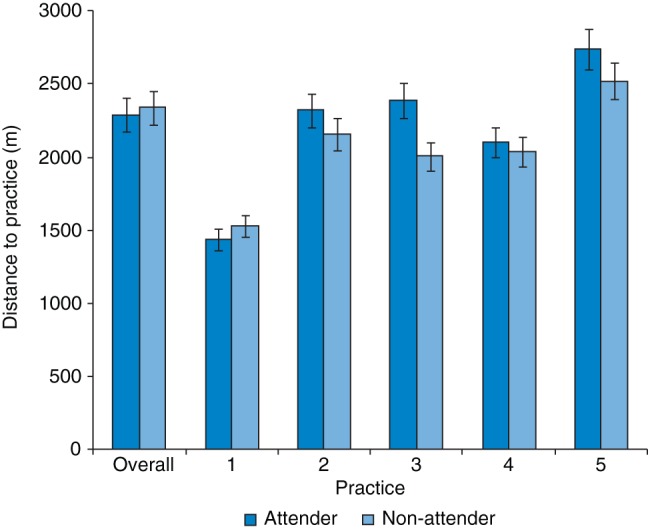
Distance to practice in attenders and non-attenders, overall and by practice.

Mean distance to practice was 2.41 ± 1.73 km (∼1.4 miles), ranging from 0 (same postcode area) to 12.3 km (∼7.7 miles). This varied significantly by practice (Kruskal–Wallis test-statistic = 333.27_(4)_, *P* < 0.001), but not between attenders and non-attenders (Mann–Whitney U = 2 633 331, Z = −1.41, *P* = 0.157; Fig. 2).

### Predictors of attendance/non-attendance

The two-stage binary logistic regression output is summarized in Table 2. In Step 1, both practice and method of invitation were significantly related to attendance and accounted for most of the variance explained following subsequent addition of the patient characteristics in Step 2 (equivalent Nagelkerke *R*^2^ change of 0.04). Aside from the between-practice variation, factors that were independently and positively related to uptake were: use of verbal/telephone invitations; increasing age (4% increase in likelihood for every additional year); female gender (47% more likely to attend than men); residing in less income-deprived areas (e.g. 59% more likely to attend if resident in least versus most deprived quintile of neighbourhood areas). Distance to GP practice was not a significant predictor of attendance.

**Table 2:** Independent predictors of Health Check attendance from Logistic Regression

*Predictor variable*	*Step 1 (Nagelkerke *R*^2^ = 0.141)*				*Step 2 (Nagelkerke *R*^2^ = 0.181)*			
	*OR*	*95% CI*		**P**	*OR*	*95% CI*		**P**
		*Lower*	*Upper*			*Lower*	*Upper*	
Constant					0.08			<0.001
Invitation method								
Letter (ref)	1.00				1.00			
Telephone/verbal	2.69	2.13	3.39	**<0.001**	2.87	2.26	3.64	**<0.001**
Practice								
5 (ref)	1.00				1.00			**<0.001**
1	2.53	1.87	3.42	<0.001	3.05	2.22	4.20	<0.001
2	1.43	1.18	1.73	<0.001	1.61	1.31	1.98	<0.001
3	1.07	0.87	1.33	0.514	1.20	0.95	1.50	0.12
4	3.37	2.73	4.15	<0.001	3.29	2.65	4.08	<0.001
Age					1.04	1.03	1.04	<0.001
Sex								
Male (ref)					1.00			
Female					1.47	1.30	1.68	**<0.001**
Income deprivation								
Q1—most deprived (ref)					1.00			**0.008**
Q2					1.11	0.87	1.43	0.395
Q3					1.24	1.03	1.49	0.022
Q4					1.30	1.06	1.61	0.014
Q5—least deprived					1.59	1.23	2.05	0.000
Distance to GP practice				1.00	1.00	1.00	0.849	

OR, odds ratio; 95% CI, 95% confidence interval.

## Discussion

### Main findings

Mean Health Check attendance in this sample of practices (61.6%) was above the city and national averages (50.2 and 49%, respectively at the time of this analysis). Age and gender biases in uptake were consistent with patterns from other general screening programmes and published Health Check data; uptake was lower in men and younger age groups (under-50s in this case),^6^ but without clear differences by ethnic group. Individuals invited to a Health Check using a telephone/verbal approach were almost three times more likely to attend than those invited by letter only, independent of other predictors. Significant differences in practice uptake remained, but this did not appear to be due to practice size within this small sample (which was excluded from analysis because of collinearity with the Practice variable). Living in less-deprived areas was associated with higher uptake, whereas living closer to the general practice (where all Health Checks were undertaken) was not associated with uptake.

### What is already known on this topic

Uptake of screening programmes is subject to various biases. Some are commonly observed across programmes, but differences in patterns and predictors will result from differences in characteristics of the programme (e.g. invasiveness of screening process, perceived risk or severity of disease), the target population and the local population. Although data are limited to five practices, the relatively large sample size, and variation in practice size (2800 to over 10 000), Health Check invitations (within- and between-practices) and a degree of geographical spread within the sample, our analyses add a dimension to evidence around uptake in relation to NHS Health Check invitation method and geographical accessibility in urban areas.

### What this study adds

The positive relationship between verbal or telephone invitations and uptake was striking. There is evidence of poor uptake from CVD health screening initiatives that rely on postal invitations,^14–16^ and some evidence for the merits of outreach work that attempts to reach those not typically engaged.^16,21^ A compromise that is perhaps more personal than a mail-out, but less resource intensive than outreach work, is to invest in telephone and in-practice opportunistic verbal recruitment. Although practice size was not a useful predictor in this sample, it is likely that telephone/verbal recruitment is more feasible in smaller practices (as inferred by Cooper and Dugdill).^6^ Our data suggest that this approach should be considered more widely.

Physical distance or proximity to the service has been shown to influence attendance in other screening programmes.^11,12^ We found no such relationship between uptake and geographical proximity of the Health Check location. Similar analysis using data from a range of rural and urban practices might produce different findings (over 90% of our sample resided in urban areas). Moreover, we could not fully capture the overall *convenience* of attending, of which distance to the service is likely to be one component. Other constraints, such as timing of appointments could be contributors. In this sample of practices only Practice 2 offered out of hours Health Check appointments on one evening each week (until 7 p.m.), but there is a range of other delivery or practice factors that could explain the considerable between-practice variance in uptake observed here and in similar analysis of Health Checks from two areas of London.^9^ These could be explored using quantitative process data from a larger sample and qualitative data to gain more in-depth insight.

### Limitations

A number of limitations are acknowledged. First, this is a small sample of practices within urban areas, which limits generalizability beyond this context. The small number of practices also precluded multilevel analysis; rather ‘practice’ was included as a predictor variable in regression analysis to identify differences in the likelihood of attendance of individuals from different practices that was not explained by invitation method. Second, it is possible that bias was introduced through selecting practices able to meet project requirements. However, these were not deemed particularly onerous and the financial incentive was offered to promote broader participation, reducing the risk of the most engaged and best performing practices self-selecting. The range of uptake rates across practices indicated a degree of success. Third, given the different record keeping across practices, it was not possible to consistently differentiate between non-responders and non-attenders, which were, therefore, treated as one group (i.e. failure to take up the invitation). Finally, practice size was considered, but excluded from the model due to collinearity with the Practice variable.

## Conclusions

Within this predominantly urban cohort, geographical proximity to the Health Check location was not an important predictor of uptake. Use of verbal or telephone invitations did emerge as a strong positive predictor of attendance and should be considered as a way to improve Health Check uptake where postal invitations are typically used. Data presented provide further evidence for commissioners and deliverers of Health Checks around *who* does not attend, and suggest that a relatively simple change to recruitment methods could increase uptake. Qualitative data from non-attenders and quantitative process data to capture important differences in recruitment and delivery would help to understand reasons for non-attendance. This could help to explain the significant between-practice variation observed here and elsewhere.

## Ethics

The study was approved by the Staffordshire University Faculty of Health Sciences Ethics committee.

## Authors’ contributions

All authors contributed to this study: C.G., N.E. and J.K. made substantial contributions to conception and design. C.G., N.E, J.R., L.C. and G.S. completed all empirical work (data collection, analysis and interpretation). All authors were involved in drafting the original manuscript and revising the manuscript critically for important intellectual content. All authors read and approved the final manuscript to be published.

## Funding

This work was funded by Stoke-on-Trent Public Health Directorate. The authors are responsible for the study design, analysis and interpretation of data, writing of the report and the decision to submit the paper for publication.

## Conflict of interest

Two of the authors were employees of the funding organisation at the time of the study (ZI, JK). Both were involved in the study conception and contributed to the manuscript, but did not influence data analysis or interpretation.
